# Dynamic Model for a Uniform Microwave-Assisted Continuous Flow Process of Ethyl Acetate Production

**DOI:** 10.3390/e20040241

**Published:** 2018-04-02

**Authors:** Yuanyuan Wu, Tao Hong, Zhengming Tang, Chun Zhang

**Affiliations:** 1 College of Information Science & Technology, Chengdu University of Technology, Chengdu 610000, China; 2 School of Electronic Information Engineering, China West Normal University, Nanchong 637002, China; 3 College of Electronics and Information Engineering, Sichuan University, Chengdu 610065, China

**Keywords:** ethyl acetate, flow processing, microwave heating, heating uniformity

## Abstract

The aim of this work is to present a model of a reaction tube with cross structures in order to improve ethyl acetate production and microwave heating uniformity. A commercial finite element software, COMSOL Multiphysics 4.3a (Newton, MA, USA), is used to build the proposed model for a BJ-22 rectangular waveguide system. Maxwell’s equations, the heat conduction equation, reaction kinetics equation and Navier-Stokes equation are combined to describe the continuous flow process. The electric field intensity, the temperature, the concentration of water, the coefficient of variation (*COV*) and the mean temperature at different initial velocities are compared to obtain the best flow rate. Four different initial velocities are employed to discuss the effect of flow velocity on the heating uniformity and heating efficiency. The point temperatures are measured by optical fibers to verify the simulated results. The results show the electric field intensity distributions at different initial velocities have little difference, which means the initial velocity will have the decisive influence on the heating process. At lower velocity, the *COV* will be smaller, which means better heating uniformity. Meanwhile, the distance between each cross structure has great influence on the heating uniformity and heating efficiency, while the angle has little. The proposed model can be applied to large-scale production of microwave-assisted ethyl acetate production.

## 1. Introduction

As an important industrial product, ethyl acetate has been widely used as solvent and intermediate [1]. Generally, ethyl acetate is produced through the Fischer esterification reaction of acetic acid [2], the Tishchenko reaction of two acetaldehyde molecules [3] and the addition of acetic acid to ethylene [4] by conventional heating, which has the limitations of energy loss and being unfriendly to the environment [5,6,7]. Microwaves, as a new kind of heating resource, have the advantages to solve the above issues caused by the conventional heating methods. For instance, the combination traditional means of drying with microwave technology can reduce processing time and produce higher quality foods in the food industry [8,9]. Microwave sintering could reduce time and energy during sintering process and improve mechanical properties and microstructure of products compared to conventional sintering process [10,11]. Microwave heating can provide a rapid, green, clean and low-cost method in biomass production [12,13]. However, during the large-scale industrial application, non-uniform heating always happens, which may lead to the burning of samples [14,15].

Many efforts have been made to improve the heating uniformity during microwave processing. The commonly used method to improve heating uniformity is mode stirrer [16,17]. The mode stirrer can change the electric field distributions and the mode of electromagnetic field in multi-mode cavities (such as microwave oven), achieving more uniform electric field distribution. Although the mode stirrer has the advantages of low cost, it is difficult to be widely used in large-scale industrial application. A phase shifting method is also proposed to improve the heating uniformity in single-mode cavities (such as waveguide) by changing the position of sample or short plane of the waveguide [18,19]. However, this method is more complicated and may cost much more than the mode stirrer method. Another popular method to overcome the non-uniform heating issue is the continuous flow process, which can be easily realized. N. G. Patil proposed a continuously operated microwave heated millireactor model in which the diameter of the reaction tube was 3 mm [20,21]. However, the production rate would be poor in this model since the flux could not be large enough in a millireactor.

In this paper, a model of reaction tube with cross structures for improving ethyl acetate production rate and microwave heating uniformity is proposed based on the multi-physics simulation of a single mode cavity operating at 2450 MHz. In this model, Maxwell’s equations, heat conduction equation, reaction kinetics equation and Navier-Stokes equation are coupled to describe the continuous flow process. The relative permittivity of the reaction mixture is expressed by the temperature and concentration of the reagents. In order to verify the model, the temperature measurement is carried out. In addition, the optimal flow rate and the parameters of the cross structures (including the angle and distance between each structure) are also discussed to achieve large-scale production and the best heating uniformity.

## 2. Methods

### 2.1. Ethyl Acetate Synthesis

In this paper, ethyl acetate is produced by the esterification of ethanol and acetic acid. The volume ratio of ethanol to acid is 1:1 and the mass fraction of sodium hydrogen sulfate, which is regarded as catalyst, is 2% of the whole reaction solution [22]. The esterification reaction is selected since the kinetics of the chemical reaction is well investigated and this process is easy to simulate in the COMSOL Multiphysics. The reaction equation can be expressed as

(1) $$CH_{3}COOH+C_{2}H_{5}OH\overset{k_{1}}{\underset{k_{-1}}{⇌}}CH_{3}COOC_{2}H_{5}+H_{2}O,$$

Acetic acid, ethanol and sodium hydrogen sulfate are provided by the local suppliers (Chengdu Kelong Chemical Reagent Factory and Chengdu Changlian Chemical Reagent Co., Ltd., Chengdu, China).

### 2.2. Experimental Setup

**Figure 1:**
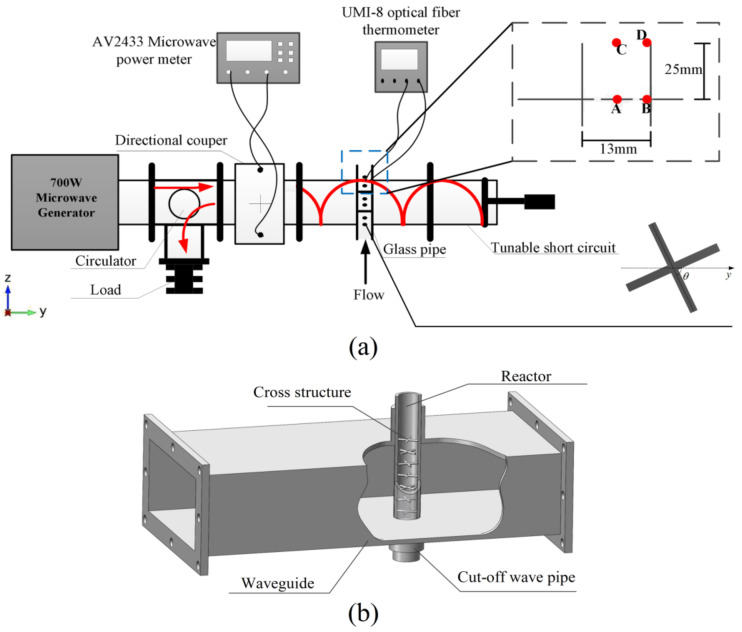
The schematic diagram of experiment system. (**a**) The 2D description of the whole system; (**b**) The 3D geometry of the waveguide and the reaction tube.

Based on the BJ-22 rectangular waveguide, the experiment system of the microwave-assisted continuous flow process is shown in Figure 1. A circulator is employed to protect the microwave generator with the output power of 700 W at 2450 MHz [23]. As a real-time monitor, an AV2433 microwave power meter is used to detect the output power of the microwave generator. A glass tube (13 mm inner diameter and 17 mm outer diameter) with eight cross structures inside is inserted through a BJ-22 waveguide (109.2 × 54.6 mm) along the center line. Each cross structure consists of two cylinders with the diameter of 1.2 mm and the center distance of adjacent cross structure is 8 mm. The top view of the bottom cross structure is also shown in Figure 1, in which the angle θ between the structure and *y*-axis is 60°. The upper cross structure can be obtained by the lower one rotating counterclockwise by 30°. The 3D rendering of the waveguide with the reactor is shown in Figure 1b. Two optical fibers connected to a FISO UMI-8 optical fiber thermometer are used to measure the temperature of two points (A and B) inside the tube, as shown in Figure 1. Sites A and C are in the center of the tube while sites B and D nears the inner wall. The tube is placed at a proper location with the highest electric field intensity in the waveguide by adjusting the short circuit. The well mixed reactants flow into the tube from the bottom and four different flow rates are employed, including 4 cm/s, 6 cm/s, 8 cm/s and 10 m/s.

### 2.3. Calculation Model

**Figure 2:**
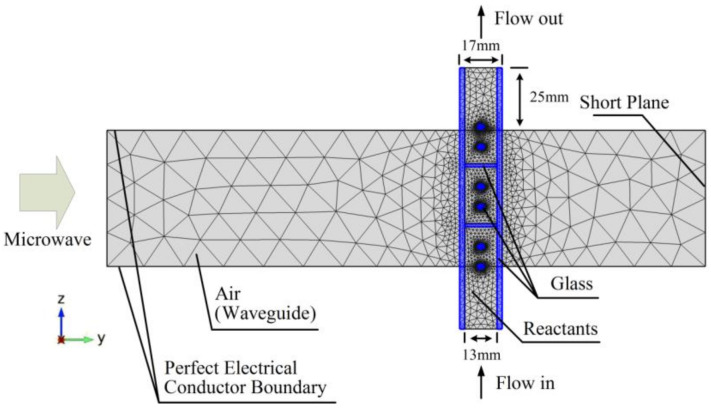
The 2D cross-section diagram of calculation model.

A 3D geometry of the experiment system is built with the commercial finite element software, COMSOL Multiphysics 4.3a (Newton, MA, USA). The 2D cross-section diagram of the geometry is shown in Figure 2. Five of the six faces of the waveguide were set as Perfect Electric Conductors (PEC) except the left-side face as shown in Figure 2 [23]. Microwaves come into the waveguide from the left-side plane. The short plane has been adjusted in order to get the strongest electric field intensity in the reactants. The glass tube has been assumed to be a no loss material, that is, its dielectric loss factor is zero but it has a dielectric constant value of 4.2. The electromagnetic waves module and laminar flow module are calculated in the frequency domain and the chemical reaction module and the heat transfer module are calculated in the time domain. The electromagnetic field will be updated every 1.5 s and the temperature will be updated in real time.

### 2.4. Relative Permittivity

For the reaction mixture, the relative permittivity can be expressed as a function of the temperature and concentration of the reagents by Gaussian function [24]. The formulas of the real part and imaginary part of relative permittivity are as follows [25]

(2) $$\varepsilon_{r}^{\prime }=3.88228+130.41841*e^{-\frac{1}{2}{\left(\frac{C_{H_{2}O}-11.330818}{3.4761753}\right)}^{2}}+16.605825*e^{-\frac{1}{2}{\left(\frac{T-367.197194}{77.776514}\right)}^{2}}$$

(3) $$\varepsilon_{r}^{\prime \prime }=23.691687-17.074037*e^{-\frac{1}{2}{\left(\frac{C_{H_{2}O}-0.2996476}{6.8177709}\right)}^{2}}-0.52576253*e^{-\frac{1}{2}{\left(\frac{T-335.305924}{7.2750168}\right)}^{2}}$$

where $C_{H_{2}O}^{}$ is the concentration of water in the reaction mixture (mol/L) and T is the absolute temperature. It is worthy of note that the relative permittivity of the reaction mixture depends on both the chemical process and temperature.

### 2.5. Thermal Model

The temperature is determined by the heat conduction equation describing the heat balance in the experiment system

(4) $$\rho_{m}C_{p}(\frac{\partial T(\vec{r},t)}{\partial t}+\vec{u}\cdot \nabla T(\vec{r},t))=K_{t}\nabla^{2}T(\vec{r},t)+P_{d}(\vec{r},t)$$

where $\rho_{m}$ is the effective density, $C_{p}$ is the effective specific heat, $K_{t}$ is the effective thermal conductivity, $\vec{u}$ is the fluid velocity vector and $P_{d}$ is the electromagnetic power dissipated per unit volume, which can be expressed by

(5) $$P_{d}(\vec{r},t)=\frac{1}{2}\omega \varepsilon_{0}\varepsilon_{r}^{\prime \prime }{\left|\vec{E}\right|}^{2}$$

where $\varepsilon_{0}$ is the permittivity of vacuum, ω is the angular frequency and $\vec{E}$ is the electric field. Generally, the effective density, effective thermal conductivity and effective specific heat of the reaction mixture are regarded as functions of mass ratios of reagents, which can be found in Table 1 [26]. The density and the specific heat capacity are set as constant due to the little variation at different temperature. Besides, the energy exchanges due to the reaction are not considered, since the energy due to the microwave heating is far higher than that due to the reaction.

### 2.6. Flow Model

Since the Reynolds number is small, the laminar flow is considered to describe the flow property in this model. The flow of the reaction mixture can be expressed by the Navier-Stokes momentum balance equation [27]

(6) $$\rho_{m}(\frac{\partial \vec{u}}{\partial t}+\vec{u}\cdot \nabla \vec{u})=-\nabla P+\hat{u}\nabla^{2}\vec{u}$$

and the mass conservation from continuity equation

(7) $$\nabla \vec{u}=0$$

where $\hat{u}$ is the effective dynamic viscosity and P is the pressure. The data of the dynamic viscosity can be seen in Table 1.

### 2.7. Chemical Reaction Kinetics

The ethyl acetate production by ethanol and acetic acid follows a second-order reversible kinetics which can be expressed as reaction kinetics

(8) $$-\frac{d(C_{RCOOH}^{0}-C_{H_{2}O}^{})}{dt}=k_{1}(C_{RCOOH}^{0}-C_{H_{2}O}^{})(C_{C_{2}H_{5}OH}^{0}-C_{H_{2}O}^{})-k_{-1}C_{H_{2}O}^{2}$$

where $C_{RCOOH}^{0}$ and $C_{C_{2}H_{5}OH}^{0}$ are the initial concentrations of acetic acid and ethanol respectively and the forward reaction rate constant $k_{1}$ and reverse reaction rate constant $k_{-1}$ can be expressed by the Arrhenius equation

(9) $$k_{1}=A_{1}\exp(-\frac{E_{a}^{f}}{RT})$$

(10) $$k_{-1}=A_{-1}\exp(-\frac{E_{a}^{r}}{RT})$$

where $E_{a}^{f}$ and $E_{a}^{r}$ are the activation energy of the forward and reverse reaction with the values of 45.28 kJ/mol, the pre-exponential factor $A_{1}$ and $A_{-1}$ are 0.68 m^3^/(mol s) and 0.17 m^3^/(mol s) and R is universal gas constant [22].

### 2.8. Mesh Size

In order to get accurate simulation results, the mesh size of the proposed model should be determined. In the simulation of microwave heating static materials, at least 10 cells per wavelength are needed to get the accurate results [28]. In this model, the heated materials are the flowing mixtures of reactants. Hence, the normalized power absorption (NPA), which is the ratio of the simulated average dissipated power of the processed medium to the effective input power, is used. When the value of NPA almost does not change with thinner mesh sizes, it can be concluded that the convergence has been reached and the results with the mesh size are accurate. In this way, the mesh size used in this paper is defined as

(11) $$m_{meshsize}\leq \frac{c}{10f\sqrt{\varepsilon_{r}}}$$

where *c* is the velocity of light and *f* is the microwave frequency.

### 2.9. Boundary Condition

As shown in Figure 2, the microwave energy comes into the waveguide from the left side. And the other faces are set as PEC, except the outlet and inlet of the reaction tube [23]

(12) $$\vec{n}\times \vec{E}=0,\;\vec{n}\times \vec{H}=0$$

where $\vec{n}$ is the unit vector perpendicular to the interface and $\vec{B}$ is the magnetic flux density vector.

The initial temperature of reaction mixture flowing into the tube is set as 303.15 K. In order to simplify the model, the reaction mixture will not exchange heat with the reaction tube, the boundary condition can be expressed as

(13) $$-k\frac{\partial T}{\partial n}=0$$

Besides, a hydrodynamic no-slip boundary condition is used to describe the laminar flow [29]. The bottom of the reaction tube is set as inlet and the constant velocities are set as 10 cm/s, 8 cm/s, 6 cm/s and 4 cm/s in the model and experimental. The top of the tube is set as outlet.

## 3. Results and Discussion

**Figure 3:**
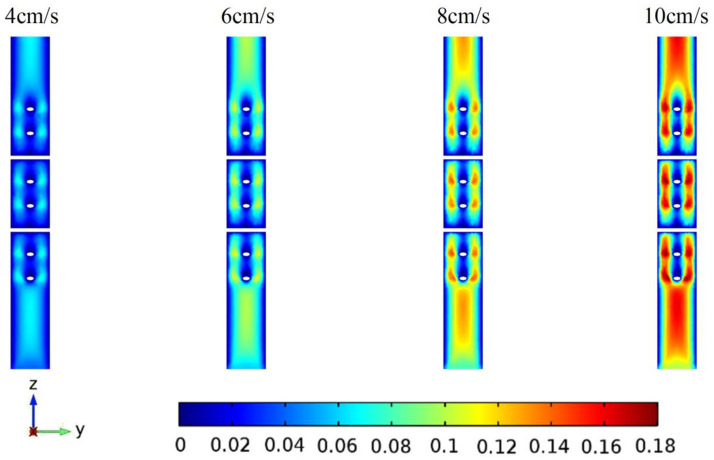
The flow velocity profiles (m/s) of the Y-Z cross section at different initial flow velocities.

The reaction mixture flows into the tube from the bottom of the pipe, which will be heated by the microwaves. The velocity profiles of reactants in Y-Z plane (X = 54.6 mm) are shown in Figure 3 at different initial velocities. As can be seen in Figure 3, there exists blanks in each profile due to the cross structures. Meanwhile, the velocity uniformity is much better than that without the cross structures, since the friction between the glass and the solution is larger than that in the solution, resulting in a fully developed laminar flow model.

**Figure 4:**
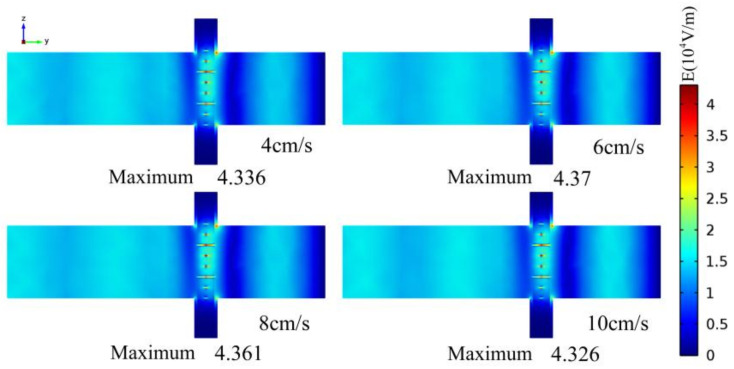
Electric filed intensity profile of the Y-Z cross section of the whole calculation zone at time 3 s.

Figure 4 shows the electric field intensity profiles of the whole calculation zone in Y-Z plane at time 3 s with different initial velocities. The electric field intensity distributions at different initial velocities have little difference and the maximum ones are nearly the same and distribute in the cross structures due to the tremendous difference of relative permittivity between reactants and glass. The results indicate that the effect of the relative permittivity on the temperature will be almost the same in this continuous flow processing and the initial velocity will have the decisive influence on the heating process.

**Figure 5:**
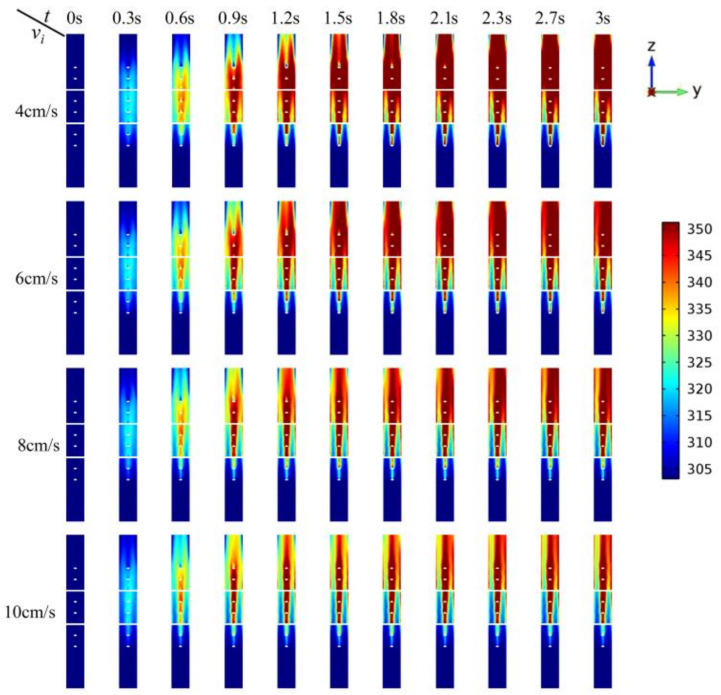
The Y-Z cross section (X = 54.6 mm) temperature profiles (K) at different time and initial flow velocities.

**Figure 6:**
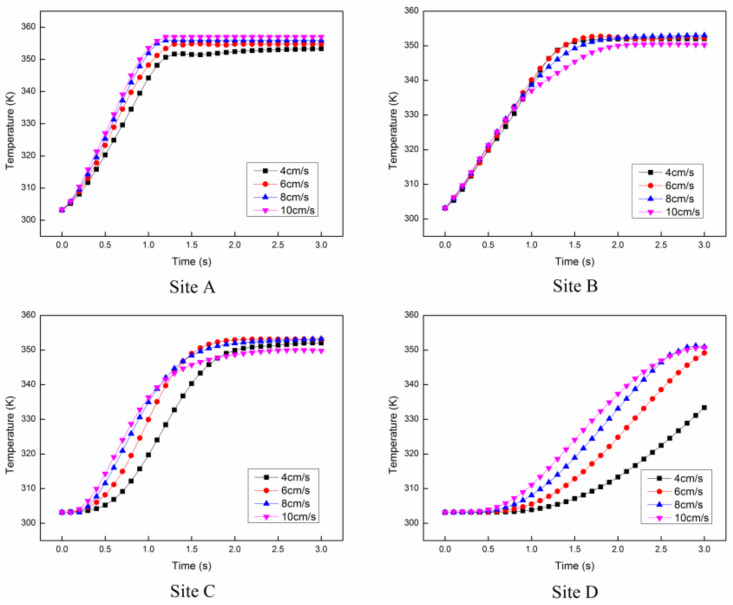
The temperature rises of the 4 sites at different initial flow velocities.

The temperature distributions of the reactants at the Y-Z cross section (X = 54.6 mm) are shown in Figure 5 from 0 s to 3 s. At different initial velocities, the temperature of the reactants at the center of the pipe stays steady after 1.2 s, since the electric field intensity at the center of the pipe is relatively stronger. For the area with lower electric field intensity, the time for temperature to be steady will be longer. For instance, the temperature near the outlet of the pipe stays constant till 3 s. Figure 6 shows the temperature rise at 4 different sites inside the tube as indicated in Figure 1. The results show that the temperatures of site A and site B have the similar trend, even though one is at the center of the pipe and the other is near the wall. This is because site A is near the cross structure and site B is neighboring the wall, resulting in relatively slower velocity. Meanwhile, the temperatures of site C and site D have a huge difference, since site C is no longer among the cross structures, leading to a faster velocity than site D.

It is worthy of note that the temperature validation is not easy, since the thermal image of the cross section is impossible to be photographed due to the semi-closed reaction tube. However, the point temperature can be measured since the reaction mixture is flowing in a steady mode. Once the optical fiber is small enough and inserted from the outlet, which will not affect the flowing mode, the point temperature can be measured. Meanwhile, the measure point is above the waveguide and the flow mode of the reaction mixture in the waveguide will not be affected. The simulation results show that the temperatures at 3 s of site A and site B are 353.1 K and 351.9 K (flow rate: 4 cm/s), 354.44 K and 352.02 K (flow rate: 6 cm/s), 355.59 K and 353.05 K (flow rate: 8 cm/s) and 356.20 K and 350.01 K (flow rate: 10 cm/s), which are shown in Table 2. The experiment is carried out to verify the simulation after 3 s which is also shown in Table 2. The results show that maximum relative error is 1.769%. However, the maximum absolute error is about 6 K, since the boiling point is about 351 K in the experiment. Although the microwave dissipation power is set as zero when the temperature is larger than 351.15 K, the heat transfer is calculated in real time and the reaction solution is heat by microwaves which is updated every 1.5 s, this may lead to the higher temperature than the boiling temperature.

**Figure 7:**
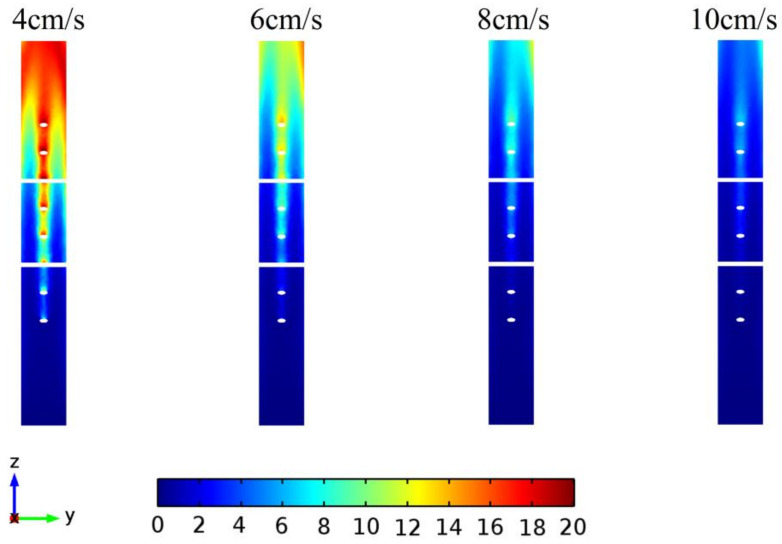
The concentrations of water (mol/m^3^) at 3 s with different flow velocities.

In this model, the concentrations of water (Y-Z cross section with X = 54.6 mm) at different velocities at 3 s are shown in Figure 7. It can be seen that the concentration of water at lower velocity is higher at the outlet of the pipe than that at higher velocity. Besides, the concentration of water at the center of the pipe is higher than that near the wall and the cross structures. This is mainly because of the low velocity of the solution near the wall and the cross structures, resulting in more heating time and higher temperature.

**Figure 8:**
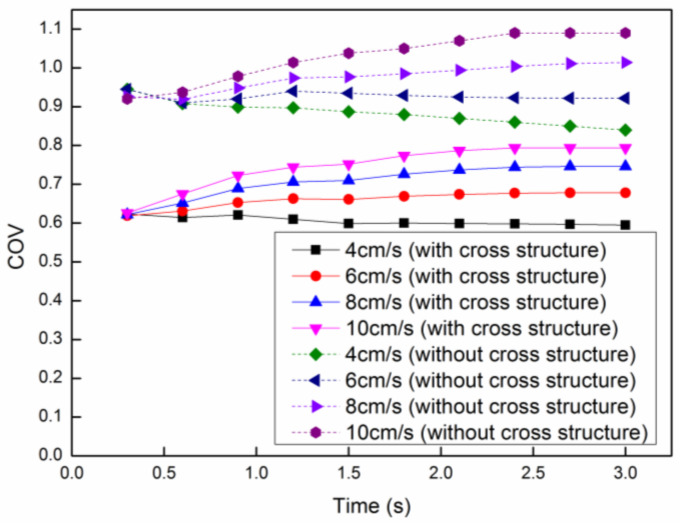
The coefficient of variation (*COV)* at different initial flow velocities.

**Figure 9:**
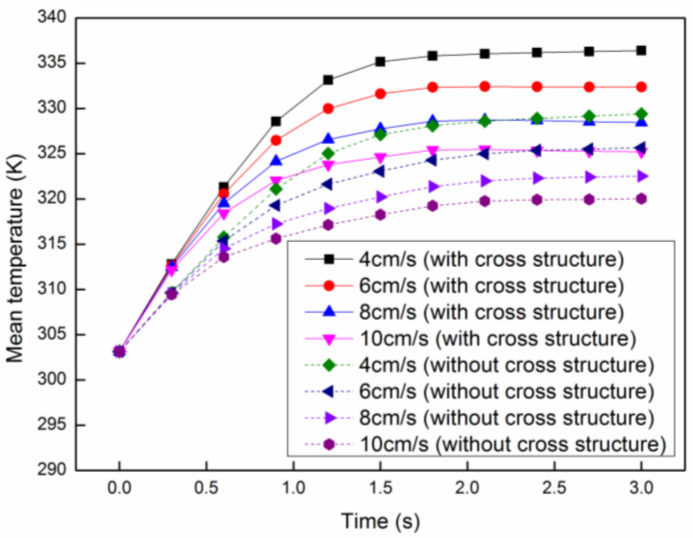
The mean temperature at different initial flow velocities.

In order to analyze the heating uniformity of the continuous flow process, the coefficient of variation (*COV*) is employed to characterize the heating uniformity. The *COV* is the ratio of the standard deviation to average, which can be expressed as

(14) $$COV=\frac{\sqrt{\frac{1}{N}\sum_{i=1}^{N}{\left(T_{i}-T_{av}\right)}^{2}}}{T_{av}-T_{0}}$$

where N is the total number of the temperature points, $T_{i}$ is the temperature at a point, $T_{av}$ is the average temperature and $T_{0}$ is the initial temperature. Generally speaking, the smaller the *COV* of the temperature, the smaller the dispersion degree of the temperature distribution, which means the better heating uniformity. The *COV* of the proposed model and the one without cross structures at each velocity is shown in Figure 8, in which the *COV* at the lower velocity is smaller than that at higher velocity in both models. On the other hand, the *COV* of the current model is much better than that of the one without cross structure at every velocity, which means the heating uniformity has been improved signally. Meanwhile, the mean temperature in both models at each velocity can be seen in Figure 9, in which the mean temperature decreases with the increasing velocity in both models. This is mainly because the reaction mixture at lower velocity will be heated by microwaves with much more time. Similar to Figure 8, the mean temperature of the current model is much higher than that of the previous model at every velocity. The mean temperature at the velocity of 8 cm/s in the current model is approximate to that at the velocity of 4 cm/s in the model without cross structure, which means the production rate and heating efficiency can be greatly improved in the current model. By considering the heating uniformity and heating efficiency, the best flow rate is 4 cm/s.

**Figure 10:**
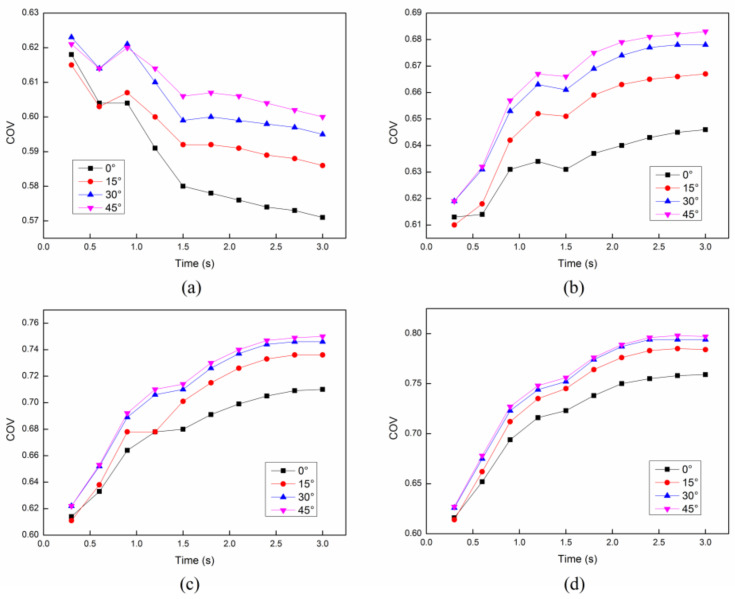
The *COV* with different angles between each structure at different initial flow velocities: (**a**) 4 cm/s; (**b**) 6 cm/s; (**c**) 8 cm/s; (**d**) 10 cm/s.

**Figure 11:**
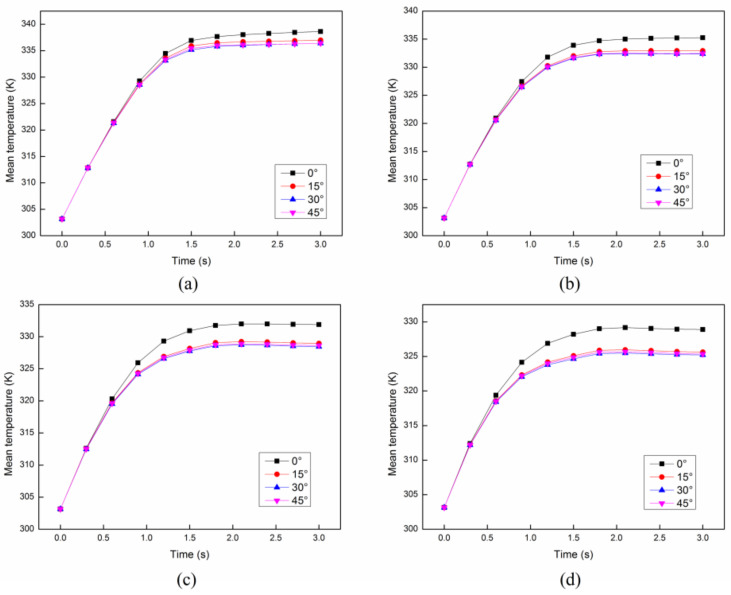
The mean temperature with different angles between each structure at different initial flow velocities: (**a**) 4 cm/s; (**b**) 6 cm/s; (**c**) 8 cm/s; (**d**) 10 cm/s.

**Figure 12:**
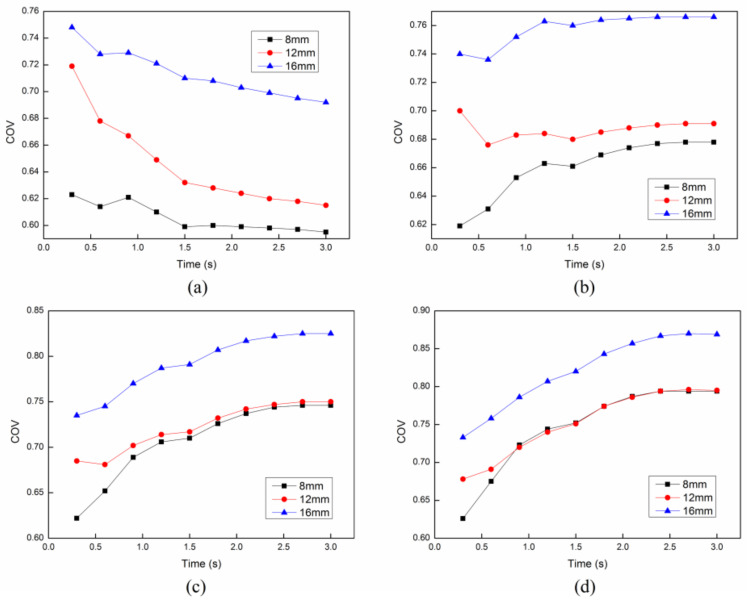
The *COV* with different distances between each structure at different initial flow velocities: (**a**) 4 cm/s; (**b**) 6 cm/s; (**c**) 8 cm/s; (**d**) 10 cm/s.

**Figure 13:**
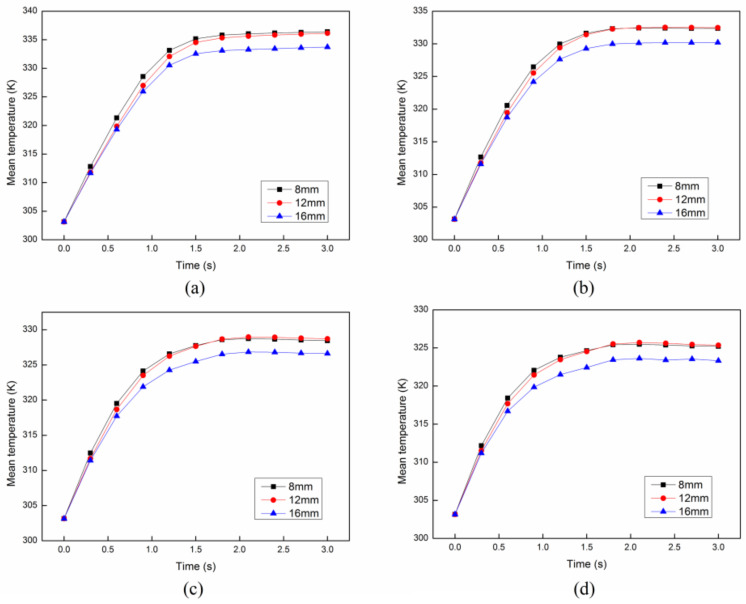
The mean temperature with different distances between each structure at different initial flow velocities: (**a**) 4 cm/s; (**b**) 6 cm/s; (**c**) 8 cm/s; (**d**) 10 cm/s.

In order to analyze the effect of parameter setting, such as the angle and the distance between each cross structure, on the heating uniformity and heating efficiency, we have compared the results of the *COV* and mean temperature in the cases of different angles and distances. Firstly, the distance between each cross structure is set as constant 8 mm. The *COV* and mean temperature at different initial velocities with different angles between each structure are compared as shown in Figure 10 and Figure 11. It can be easily concluded that the angle between each cross structure has a little effect on the heating uniformity. On the other hand, the heating efficiency can be only improved with the smallest angle 0°. Secondly, the angle between each cross structure is set as constant 30°. The *COV* and mean temperature at different initial velocities with different distances between each structure are compared as shown in Figure 12 and Figure 13. It is obvious that the distance between each cross structure has great influence on the heating uniformity and heating efficiency. The heating uniformity and the heating efficiency are much better with shorter distance. However, at each velocity, the heating uniformity and the heating efficiency for the distance of 8 mm and 12 mm are nearly the same, which means the heating uniformity and the heating efficiency are approaching a steady state with the shorter distance.

## 4. Conclusions

In this paper, a model of a reaction tube with eight cross structures is proposed to improve ethyl acetate production rate and microwave heating uniformity. In this model, the best flow rate for the continuous flow process is 4 cm/s when the angle and the distance between each cross structure are 30° and 8 mm respectively, by considering both heating uniformity and heating efficiency. Besides, the tube with cross structures can obviously improve the heating uniformity and mean temperature compared to the tube without cross structures, due to the 10 K higher mean temperature and about 0.25 smaller *COV* at each flow velocity. Meanwhile, we find that the angle between each cross structure has little influence on the heating uniformity and heating efficiency, especially for the larger angle. The case with an angle of 0° has relative better *COV* and mean temperature and the other three cases have nearly the same *COV* and mean temperature, while the case with a shorter distance between each cross structure has much better heating uniformity and heating efficiency. This model can be used to deal with the thermal analysis during the microwave assisted production of ethyl acetate. Furthermore, the inhomogeneous heating can be avoided by an appropriate flow velocity or distance between each cross structure.

## Figures and Tables

**Table 1:** The thermal properties used in the model.

Parameter		Equation
Thermal conductivity (W/m·K)	Ethanol	0.2525 − 0.00028 × T
	Acetic acid	0.2128 − 0.000184 × T
	Ethyl acetate	0.2513 − 0.00036 × T
	Water	0.1896 + 0.0014 × T
Density (kg/m^3^)	Ethanol	789.3
	Acetic acid	1049.2
	Ethyl acetate	900
	Water	1000
Specific heat capacity (J/kg·K)	Ethanol	2440
	Acetic acid	2055
	Ethyl acetate	1940
	Water	4200
Dynamic Viscosities (Pa·s)	Ethanol	0.01809 − 0.00009572 × T + 0.0000001296 × T^2^
	Acetic acid	0.009403 − 0.0000441 × T + 0.000000054 × T^2^
	Ethyl acetate	0.004058 − 0.00001983 × T + 0.0000000256 × T^2^
	Water	0.02478 − 0.0001409 × T + 0.0000002038 × T^2^

**Table 2:** The averaged simulated and measured temperature results at the 2 sites under different initial flow velocities.

Initial Velocity (cm/s)	Site	Simulated Temperature (K)	Measured Temperature (K)	Error (%)
4	A	353.1	350.90	0.623%
	B	351.9	350.55	0.384%
6	A	354.44	350.95	0.985%
	B	352.02	350.30	0.489%
8	A	355.59	349.85	1.614%
	B	353.05	349.80	0.921%
10	A	356.20	349.90	1.769%
	B	350.01	349.75	0.074%
